# The Epidemiology of Sleep Quality, Sleep Patterns, Consumption of Caffeinated Beverages, and Khat Use among Ethiopian College Students

**DOI:** 10.1155/2012/583510

**Published:** 2012-11-25

**Authors:** Seblewengel Lemma, Sheila V. Patel, Yared A. Tarekegn, Mahlet G. Tadesse, Yemane Berhane, Bizu Gelaye, Michelle A. Williams

**Affiliations:** ^1^Addis Continental Institute of Public Health, Addis Ababa, Ethiopia; ^2^Multidisciplinary International Research Training Program, Department of Epidemiology, Harvard School of Public Health, 677 Huntington Avenue, Kresge 501, Boston, MA 02115, USA; ^3^Department of Mathematics & Statistics, Georgetown University, Washington, DC 20057, USA

## Abstract

*Objective*. To evaluate sleep habits, sleep patterns, and sleep quality among Ethiopian college students; and to examine associations of poor sleep quality with consumption of caffeinated beverages and other stimulants. *Methods*. A total of 2,230 undergraduate students completed a self-administered comprehensive questionnaire which gathered information about sleep complaints, sociodemographic and lifestyle characteristics,and theuse of caffeinated beverages and khat. We used multivariable logistic regression procedures to estimate odds ratios for the associations of poor sleep quality with sociodemographic and behavioral factors. *Results*. Overall 52.7% of students were classified as having poor sleep quality (51.8% among males and 56.9% among females). In adjusted multivariate analyses, caffeine consumption (OR = 1.55; 95% CI: 1.25–1.92), cigarette smoking (OR = 1.68; 95% CI: 1.06–2.63), and khat use (OR = 1.72, 95% CI: 1.09–2.71) were all associated with increased odds of long-sleep latency (>30 minutes). Cigarette smoking (OR = 1.74; 95% CI: 1.11–2.73) and khat consumption (OR = 1.91; 95% CI: 1.22–3.00) were also significantly associated with poor sleep efficiency (<85%), as well as with increased use of sleep medicine. *Conclusion*. Findings from the present study demonstrate the high prevalence of poor sleep quality and its association with stimulant use among college students. Preventive and educational programs for students should include modules that emphasize the importance of sleep and associated risk factors.

## 1. Introduction 

Sleep is important for maintaining good physical, mental, and emotional health [1]. Short sleep duration (generally defined as less than 7 hours) increases rates of mortality and has been reported as an important risk factor for adverse cardiovascular, endocrine, immune, and nervous system outcomes, such as obesity among adults and children, diabetes and impaired glucose tolerance, cardiovascular disease and hypertension, mood and anxiety disorders, and substance abuse [2–7]. Although there is a well-established body of evidence that has evaluated sleep among adults and children, few have investigated the prevalence and influences of poor sleep among younger, college-aged students. Furthermore, there is limited research on the influences of caffeine and the consumption of other stimulants on sleep duration and sleep quality among college-aged students and young adults.

Caffeine, the most widely used drug in the world [8], is believed to influence the performance and mental state by lack or loss of sleep [9, 10]. Some studies have reported that caffeine is beneficial in restoring low levels of wakefulness and in offsetting the reduced cognitive abilities that result from sleep deprivation [8] while other investigators have demonstrated that caffeine had a significant negative effect on mood and performance when ingested after a period of sustained abstinence [9, 10]. Caffeine consumption has also been shown to adversely impact sleep patterns in manners that promote daytime sleepiness [11]. A fairly substantial literature has documented associations of caffeine consumption, particularly high levels, with alterations in multiple indices of sleep duration and sleep-quality among students and young adults. For instance, high-caffeine users in Massachusetts were found to have shorter sleep duration, more disturbed sleep, longer sleep latencies, more complaints of daytime sleepiness, and poor sleep quality when compared with low users [12]. One study conducted in Iceland reported that adolescents use caffeine to delay sleep during nighttime [13]. However, these studies have almost exclusively been conducted in populations residing in North America, Europe, and Asia. Few studies have been conducted among populations residing in sub-Saharan Africa. Moreover, few studies have simultaneously explored participants' sleep patterns and their use of stimulants.

In light of the noted gaps in the literature and given the increased marketing and consumption of caffeinated beverages and other stimulants across the globe, particularly in low and middle income countries, we conducted this study to evaluate sleep patterns and sleep quality among Ethiopian college students. Specifically, we sought to examine the relationship between poor sleep quality and consumption of caffeinated beverages. We also sought to evaluate the extent to which khat consumption (khat is an evergreen plant with amphetamine-like effects commonly used as a mild stimulant for social recreation and to improve work performance in Ethiopia [14, 15]) among Ethiopian students is associated with altered sleep patterns and sleep quality. Findings from this study will help identify factors influencing poor sleep and will provide objective evidence that may then be used to guide the development of health and wellness programs for this population.

## 2. Methods

### 2.1. Study Setting and Sample

A cross-sectional survey was conducted at the Universities of Gondar and Haramaya, Ethiopia. The University of Gondar is located in the city of Gondar in the northern part of Ethiopia. It is one of the largest Universities in the country. Currently there are six faculties with 11,000 regular and 5,000 extension students in 35 undergraduate and 8 graduate programs. Haramaya University (formerly known as Alemaya) is located about 510 kms south of Addis Ababa in the Eastern Hararghe Zone. Haramaya University is a self-contained University village with a total of 13,823 undergraduate students who are enrolled across thirteen colleges, faculties, and schools. The two universities were selected based on their large number of students and their willingness to participate in the study. They also represent two different sociocultural settings, Haramaya University is located in a traditionally khat growing area while Gondar University is in an area where khat growing and consumption have substantially increased only over the last decade.

A multistage sampling design by means of probability proportional to size (PPS) was used to select departments. This approach was performed for both universities, and all students in selected departments were invited to participate. Students who expressed an interest in participating in the study were invited to meet in a large classroom or an auditorium where they were informed about the purpose of the study and asked to participate in the survey. A random sample of these students was then selected and asked to complete a self-administered individual survey after providing consent. There was no set time limit for completing the survey. Students who could not read the survey (i.e., were blind) were excluded, as were those enrolled in correspondence, extension, or night school programs. A total of 2,817 undergraduate students participated in the study. For the study described here, after excluding subjects with incomplete questionnaires and missing sleep quality scores, the final analyzed sample consisted of 2,230 students. Based on the information provided, students excluded from analysis had similar characteristics to those considered. All completed questionnaires were anonymous, and no personal identifiers were used. Approval to conduct this study was obtained from the deans of all participating colleges. The procedures used in this study were approved by the institutional review boards of Addis Continental Institute of Public Health and Gondar University, Ethiopia and the University of Washington, USA. The Harvard School of Public Health Office of Human Research Administration, USA granted approval to use the deidentified data set for analysis.

### 2.2. Data Collection and Variables

A self-administered questionnaire was used to collect information for this study. The questionnaire ascertained demographic information including age, sex, and education level. Questions regarding behavioral risk factors such as caffeinated beverages, tobacco, alcohol, and khat consumption were also included. Participants' anthropometric measurements were taken by research nurses using standard protocols. Height and weight were measured without shoes or outerwear. Height was measured to the nearest 0.1 cm and weight was measured to the nearest 0.1 kg with subjects standing on a scale. All anthropometric values consisted of the mean of three measurements.

### 2.3. Use of Caffeinated Beverages and Khat Use

Participants were first asked if they consumed any stimulant or energy drink during the past week. Participants answering “Yes” were further asked to identify the specific type of caffeinated beverages. These included coffee, espresso, spris (coffee mixed with tea), macchiato, cappuccino, Coke, and Pepsi. For the purpose of this analysis we grouped coffee and espresso (no versus yes), macchiato and cappuccino (no versus yes), and Coke, and Pepsi (no versus yes). The questionnaire also ascertained information about khat consumption. Affirmative responses to khat use were followed with questions about frequency per week and participants' khat use was categorized as none, 1-2 times per week, and ≥3 times per week.

### 2.4. Pittsburgh Sleep Quality Index (PSQI)

Sleep quality was assessed using the previously validated Pittsburgh Sleep Quality Index (PSQI) [16]. The PSQI instrument has been validated among college students in sub-Saharan Africa [17]. The PSQI is a 19-item self-reported questionnaire that evaluates sleep quality over the past month. The PSQI yields seven sleep components related to sleep habits including duration of sleep, sleep disturbance, sleep latency, habitual sleep efficiency, use of sleep medicine, daytime dysfunction, and overall sleep quality. The sleep components yield a score ranging from 0 to 3, with three indicating the greatest dysfunction [16]. The sleep component scores are summed to yield a total score ranging from 0 to 21 with higher total scores (referred to as global scores) indicating poor sleep quality. Based on prior literature [16], participants with a global score of >5 were classified as poor sleepers. Those with a score of 5 or less were classified as good sleepers.

For sleep quality subscales, which consist of subjective sleep latency, sleep efficiency, daytime dysfunction due to sleepiness, and sleep medication use, we computed dichotomous variables of optimal and suboptimal sleep quality. Specific categories were long sleep latency (≥30 minutes versus <30 minutes); poor sleep efficiency (<85% versus ≥85%); daytime dysfunction due to sleepiness (<once a week versus ≥once per week); sleep medication use during the past month (<once a week versus ≥once per week). Sleep duration was assessed using the PSQI questionnaire that queried how many hours of actual sleep the participants got at night during the previous month. Given the lack of prior data on cutoffs for defining “short sleep duration” among college students, we used quartiles. The following quartiles were used to define sleep duration: ≤6.0 hours, 6.1–7.0 hours, 7.1–8.0 hours, and ≥8.1 hours. The group with the lowest quartile of sleep duration (≤6 hours) was defined as short duration sleepers.

### 2.5. Other Covariates

Body mass index (BMI) was calculated as weight (kg)/height squared (m^2^). BMI thresholds were set according to the World Health Organization (WHO) protocol (underweight: <18.5 kg/m^2^; normal: 18.5–24.9 kg/m^2^; overweight: 25.0–29.9 kg/m^2^; obese ≥30 kg/m^2^) [18]. We defined alcohol consumption as none (<1 alcoholic beverage a week), moderate (1–19 alcoholic beverages a week), and high to excessive consumption (>19 alcoholic beverages a week) [19, 20]. The other covariates included: age (years), sex, smoking history (never versus ever), and regular participation in moderate or vigorous physical activity (no versus yes).

### 2.6. Data Analysis

We first examined frequency distributions of sociodemographic and behavioral characteristics of study participants. Characteristics were summarized using means (±standard deviation) for continuous variables and counts and percentages for categorical variables. We also calculated the distribution of poor sleep quality across demographic and behavioral groups. Chi-square tests and Student's *t*-tests were used to determine bivariate differences for categorical and continuous variables, respectively. Tests for linear trends in proportions were also considered for ordinal variables. Confounding variables were considered *a priori* on the basis of their hypothesized relationship with sleep quality and lifestyle characteristics. We used multivariable logistic regression models to estimate odds ratios (OR) and 95% confidence intervals (95% CI) for the associations of sleep quality parameters with sociodemographic and behavioral factors. Forward logistic regression modeling procedures combined with the change-in-estimate approach were used to select the final models reported in this research [21]. Variables of *a priori* interest (i.e., age) were forced into final models. Because prior literature suggest substantial gender differences in sleep quality and patterns by sex [1], the frequency distribution of PSQI subscales were calculated separately for male and female students. Prevalence estimates were also obtained for the dichotomous sleep quality subscales in relation to stimulant drinks and lifestyle characteristics. All analyses were performed using IBM's SPSS Statistical Software for Windows (IBM SPSS Version 20, Chicago, IL, USA). All reported *P* values are two-sided and deemed statistically significant at *α* = 0.05.

## 3. Results

### 3.1. Study Sample Demographics and Behavioral Characteristics

**Figure 1:**
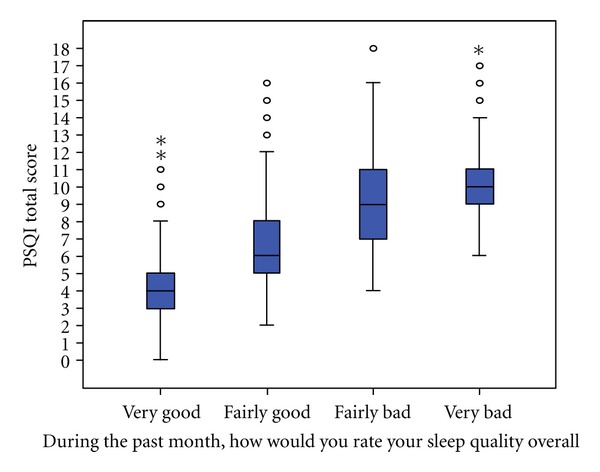
Comparison of Pittsburgh Sleep Quality Index scores and self-ratings of sleep quality.

Of 2,230 students, 1,175 (52.7%) were classified as having poor sleep quality (PSQI > 5). When comparing subjective measures of self-rated sleep quality with PSQI global scores, poor sleep quality was underreported by 41.3% of students relative to PSQI classification (Figure 1). Approximately 25% of the very good and 60% of the fairly good self-ratings for overall sleep quality had PSQI scores greater than 5. Nearly all (94.5%) of those who self-rated their sleep quality as fairly bad and all of those who self-rated their sleep quality as very bad were classified as having poor sleep quality using the PSQI scale.

Table 1 summarizes the association between demographic and lifestyle characteristics with sleep quality status as determined by the PSQI. Most participants were male students (77.3%), with a mean (SD) age of 21.6 (1.7) years, and were nonsmokers (96.2%). Nearly a fifth (19.1%) of participants reported drinking at least one alcoholic beverage per month and 10.7% reported that they currently chew khat. Notably, only 1.3% of students in this population were classified as overweight and a majority of students (67.0%) reported being physically active. Among these characteristics, BMI and physical activity were found to have statistically significant association with sleep quality status.

### 3.2. Sleep Quality Subscales Stratified by Sex

**Figure 2:**
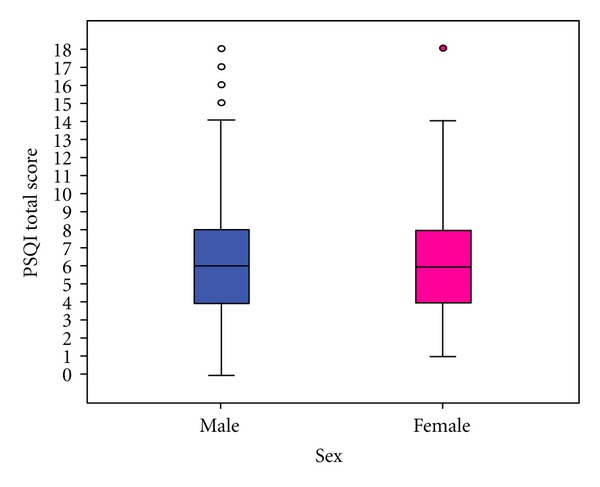
Distribution of Pittsburgh Sleep Quality Index score by sex.

**Figure 3:**
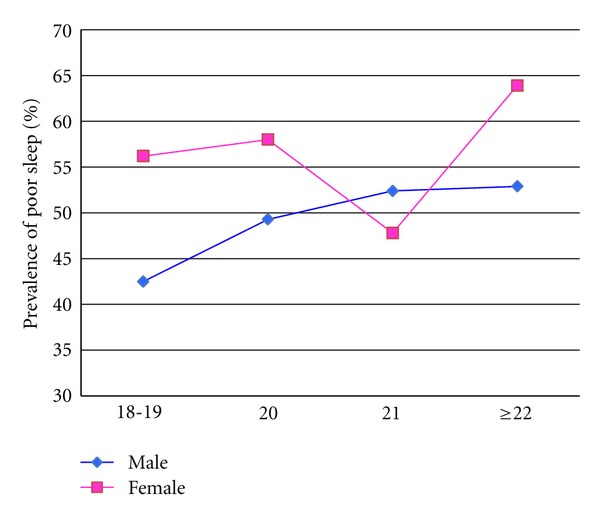
Prevalence of poor sleep quality in relation to age and sex.

A comparison of the distribution of sleep quality, according to PSQI global scores, between males and females is summarized in Figure 2. More than half of study participants (52.7% of males and 55.9% females) had global sleep scores >5, thus classifying them as poor sleepers. The prevalence of poor sleep quality in relation to age and sex is depicted in Figure 3. As males' age increases, a greater percentage experience poor sleep quality. The incidence of poor sleep quality is higher among females across all age groups, except at age 21, where it drops substantially before increasing to a maximum of 64% in the older age group (≥22 years old).

Table 2 summarizes the differences between males and females in the sleep component subscales. Female students reported short sleep duration (<6 hours) at a significantly higher frequency than males (49% versus 42%, *P* value = 0.0024). Considering some of the sleep parameters as continuous variables and using the Mann-Whitney test, there was strong evidence that female students had significantly shorter sleep duration (*P* value = 0.005) and shorter sleep latency (*P* value = 0.013). There was a marginally significant association between sex and daytime dysfunction (*P* value = 0.090). Overall, long sleep latency was reported by 48.5% of students while 3.30% reported using sleep medicine at least once per week. Additionally, 30.2% of students reported experiencing daytime dysfunction at least once a week and 30.3% reported poor sleep efficiency.

### 3.3. Odds Ratios for Poor Sleep Quality

The odds ratios for poor sleep quality across various demographic and lifestyle characteristics are reported in Table 3. In the multivariate adjusted model, the odds of poor sleep quality was 37% higher (OR = 1.37, 95% CI: 1.08–1.73) among female students when compared with male students. Consumption of one caffeine containing beverage per week and participation in any physical activity were associated with increased odds of poor sleep quality (OR = 1.29; 95% CI: 1.02–1.64) and (OR = 1.27; 95% CI: 1.02–1.64), respectively.

### 3.4. Consumption of Caffeinated Beverages and Khat Use according to Sleep Quality

**Figure 4:**
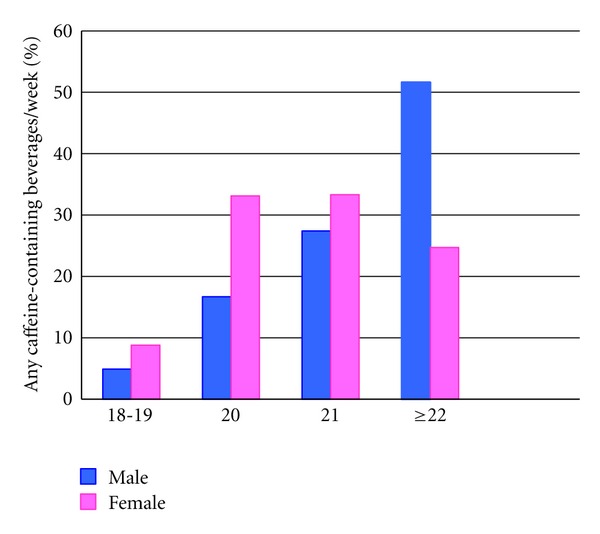
Any caffeine containing beverages consumption by age and sex.

Figure 4 shows the proportion of any caffeinated beverages consumption in relation to age and sex. Among males, consumption of caffeinated beverages appears to increase with age. Whereas among females consumption peaked among 20 year olds and eventually plateaus thereafter. The association between consumption of any caffeine containing beverages and poor sleep quality was statistically significant (*P* value = 0.015) (Table 4). Of those who experience poor sleep quality, 82.3% reported consuming some type of caffeine containing beverage. Similarly, students who reported using khat were more likely to be classified as poor sleepers than those who did not consume khat (11.9% versus 9.3%, *P* value = 0.065). We did not observe evidence of a dose response relationship between frequency of khat consumption and sleep quality (Table 4).

### 3.5. Odds Ratios for Sleep Quality Parameters in relation to Lifestyle Characteristics

We next examined associations of various parameters of sleep patterns and quality (short sleep duration, long sleep latency, daytime dysfunction, poor sleep efficiency, and sleep medicine use) with selected lifestyle characteristics including cigarette smoking, alcohol consumption, khat use, and physical activity. Results from these analyses are summarized in Table 5. Students who reported smoking cigarette compared to nonsmokers have higher odds of long sleep latency (OR = 1.68; 95% CI: 1.07–2.64), poor sleep efficiency (OR = 1.74; 95% CI: 1.11–2.73), and sleep medicine use (OR = 2.84; 95% CI: 1.26–6.43). No significant relationship was noted between alcohol consumption and sleep patterns except for sleep medicine use. High alcohol consumption (>19 drinks per month) was associated with increased odds of sleep medicine use (OR = 9.25; 95% CI: 3.53–24.2). Khat consumption, especially at a higher frequency, also increased the odds of these same parameters: long sleep latency (OR = 1.72; 95% CI 1.09–2.71), poor sleep efficiency (OR = 1.91; 95% CI: 1.22–3.00), and sleep medicine use (OR = 4.42; 95% CI: 2.06–9.47). Overall, students who reported consuming any caffeinated beverages compared to nonconsumers appeared to be at an increased risk for sleep problems, although the odds ratios did not reach statistical significance (with sleep latency being the only exception). Students who reported consuming any caffeinated beverages a week were 1.48-times as likely to report long sleep latency (OR = 1.48; 95% CI: 1.19–1.83) as compared to nonusers.

## 4. Discussion

To the best of our knowledge, this is the first study to evaluate sleep quality and stimulant use among Ethiopian college students. Approximately 53% of students in our study were classified as having poor sleep quality. Use of caffeinated beverages was reported by more than three fourth of students with poor sleepers reporting higher consumption compared to those with good sleep quality. In adjusted multivariate analyses, caffeine consumption, cigarette smoking, and khat use were all associated with increased odds of long sleep latency. Cigarette smoking and khat consumption also had a statically significant association with poor sleep efficiency and increased use of sleep medicine.

Overall, our study findings are in agreement with previous reports which indicate a high prevalence of poor sleep quality among college students [22–24]. For instance, Cheng et al. [25], in their study of Taiwanese university students, reported 54.7% were classified as happing poor sleep quality. Similarly Suen et al. [26], in study conducted in Hong Kong reported 57.5% of students were poor sleepers.Suenet al. [26], in the US, reported 42% of students were classified as poor sleepers on the PSQI scale. Collectively, despite variations in data collection methods, instruments used to classify sleep quality, as well as social, geographic, racial, and ethnic differences of populations studied to date, available evidence suggests that poor sleep quality is highly prevalent among college students across the globe and is an emerging important public health problem.

We expected caffeine consumption to be associated with increased odds of poor sleep quality subscales, but it appeared to be associated only with long sleep latency. No statistically significant association was noted between caffeine consumption and other sleep quality parameters. Our findings are in general agreement with some previous studies [27, 28]. For instance Brick et al. [27], in their study among 341 US medical students, found no significant association between caffeine use and poor sleep quality. Similarly, Lund et al. [28] in a large survey of a Midwestern university found caffeine consumption to be a nonsignificant predictor of sleep quality. It is possible that the frequency and amount of caffeine consumption impact sleep quality and its subscales [29]. Notably, investigators have shown that caffeinated beverages have a dose-dependent negative effect on sleep onset, sleep time, and sleep quality [29]. However, we did not have information concerning frequency and dose of caffeine consumption in the present study to confirm this hypothesis. Studies that carefully characterize frequency and dose of caffeine contacting beverages consumption and their long-term negative health impacts are needed.

We found that having ever smoked cigarettes increased the odds of poor PSQI sleep quality subscales. Alcohol consumption appeared to decrease the odds of experiencing short sleep duration in our study population, but greatly increased the odds of sleep medicine use. Our findings are consistent with the results of most prior studies, which support the hypothesis that unfavorable lifestyle characteristics and health risk behaviors negatively affect sleep quality [30, 31]. Similarly, in the present study, khat use was found to be associated with increased odds of long sleep latency, poor sleep efficiency, and sleep medicine use. Of the study participants who consume khat, 15.2% do so when in need of more energy in general and 45.2% chew when studying for exams or need to complete a project. Khat leaves have been consumed for many centuries for the purpose of preventing fatigue and staying alert, increasing motor stimulation, and providing a sense of excitement and energy [32, 33]. Studies have shown that khat consumption induces a state of euphoria and elation with feelings of increased alertness. However, this stage is followed by experiences of depressed mood, irritability, anorexia, and difficulty to sleep [32, 34]. It is important to note that alcohol is sometimes used to counteract the effects of khat, such as depressed mood [32]. Indeed, among khat users in our study population, there was a significantly higher prevalence of alcohol consumption. Thus, efforts to reduce the frequency of khat consumption among college students will help improve sleep quality and possibly influence alcohol consumption patterns.

The observed associations in this study are biologically plausible. When an individual requires sleep, adenosine sends fatigue signals to body cell receptors that result in an increased urge to sleep [8, 35]. Caffeine binds to cell receptors in the brain and prevents them from receiving the fatigue signal produced by adenosine, keeping individuals awake and alert [36]. Khat, which contains cathinone and cathine, induces the release of serotonin and dopamine [32]. Both increase alertness and reduce fatigue [37]. The use of these stimulants (i.e., caffeine and khat) disturbs sleep patterns and it is conceivable that chronic use may lead to reduced sleep quality and long-term adverse health effects [38].

The findings of our study should be interpreted in light of some study limitations. First, given the cross-sectional nature of our study, it is difficult to delineate whether poor sleep quality is a result of alcohol consumption, smoking status, khat and caffeine consumption, or whether these lifestyle characteristics resulted as a way to cope with the effects of poor sleep. Second, our use of a self-administered survey that relied on subjective measures of sleep quality and other covariates may have introduced some degree of error in reporting behavioral covariates, and the period of the semester when the survey was administered could have influenced the sleep quality ratings. However, we believe that these issues are in part mitigated by our use of anonymous questionnaire and validated instruments. Third, sleep quality was determined using the PSQI, which relates good sleep quality with global scores of 5 or below and poor sleep quality with scores between 6 and 21. With this grouping, there could be substantial heterogeneity among subjects deemed to be poor sleepers, potentially masking important associations. Fourth, we did not have information concerning frequency and dose of caffeinated beverages consumption in the present study. As a result, it is possible that the binary grouping of caffeinated beverages consumption attenuated the magnitude of association towards null. Finally, a well-established nonclinical treatment for sleep difficulties is regular exercise [39, 40]. However in our study, participation in physical activity was found to increase the odds of having poor sleep quality. This may be due to substantial heterogeneity among the physically active group, which may have masked the effects of different amounts of physical activity (none, low, moderate, or vigorous once or multiple times a week) on sleep quality.

In conclusion, our study provides strong evidence that poor sleep quality is highly prevalent among Ethiopian college students and demonstrates significant associations with stimulant use. This is an important contribution to research focused on adolescent and youth health. College students in Ethiopia, and possibly other parts of East Africa, should be made aware of the impact of caffeine beverage consumption and khat use on sleep quality and patterns. Improved sleep quality benefits college students in their daily activities, academic performance, and also improves their health status [22, 30, 41, 42]. A technology-filled and fast-paced society may be the reason many college students overlook the significance of adequate sleep. The college environment also provides increased exposure to sleep-inhibiting factors, like academic stress and social situations. Avoiding the build-up of a chronic sleep debt during early adulthood through awareness, education, and effective management of sleep disorders may be important in enhancing the academic performance during their college stay and in reducing the development of chronic diseases later in life.

## Figures and Tables

**Table 1:** Characteristics of the study population.

Characteristic	All **N = 2,230	Poor sleep quality **N = 1,175	Good sleep quality **N = 1,055	*P value
	*n* (%)	*n* (%)	*n* (%)	
Age (Mean ± SD)	21.6 ± 1.7	21.7 ± 1.8	21.6 ± 1.6	0.035
Age (years)				
18-19	123 (5.5)	59 (5.0)	64 (6.1)	
20	454 (20.4)	238 (20.3)	216 (20.5)	
21	627 (28.1)	321 (27.3)	306 (29.0)	0.436
22 and older	1,026 (46.0)	557 (47.4)	469 (44.4)	
Sex				
Male	1,700 (77.3)	880 (75.9)	820 (78.9)	
Female	499 (22.7)	279 (24.1)	220 (21.1)	0.103
Cigarette smoking status				
Never	2,146 (96.2)	1,126 (95.8)	1,020 (96.7)	
Ever	84 (3.8)	49 (4.2)	35 (3.3)	0.291
Alcohol consumption				
<1 drink/month	1,900 (85.2)	994 (84.6)	906 (85.9)	
1–19 drinks/month	303 (13.6)	162 (13.8)	141 (13.4)	0.168
≥20 drinks/month	27 (1.2)	19 (1.6)	8 (0.8)	
Khat consumption				
No	1,751 (89.3)	904 (88.1)	847 (90.7)	
Yes	209 (10.7)	122 (11.9)	87 (9.3)	0.065
Body mass index (kg/m^2^)				
Underweight (<18.5)	857 (38.6)	445 (38.0)	412 (39.2)	
Normal (18.5–24.9)	1,337 (60.2)	719 (61.3)	618 (58.9)	0.025
Overweight (25.0–29.9)	28 (1.2)	8 (0.7)	20 (1.9)	
Any physical activity				
No	607 (28.9)	293 (26.5)	314 (31.5)	
Yes	1,495 (71.1)	813 (73.5)	682 (68.5)	0.011

*P value from Chi-square test for categorical variables or from Student's *t*-test for continuous variables.

**Numbers may not add up due to missing data for selected variables.

**Table 2:** Pittsburgh sleep quality index (PSQI) subscales by sex.

Characteristic	All **N = 2,230	Male **N = 1,700	Female **N = 499	*P value
	*n* (%)	*n* (%)	*n* (%)	
Sleep duration (hours)				
≤6.0	979 (44.0)	718 (42.3)	245 (49.2)	0.024
6.1–7.0	409 (18.4)	311 (18.4)	91 (18.3)	
7.1–8.0	516 (23.2)	414 (24.4)	95 (19.1)	
≥8.1	321 (14.4)	253 (14.9)	67 (13.4)	
Sleep latency (minutes)				
≤15	407 (18.2)	292 (17.2)	109 (21.8)	0.117
16–30	741 (33.2)	573 (33.7)	157 (31.5)	
31–60	818 (36.7)	633 (37.2)	173 (34.7)	
≥60	264 (11.9)	202 (11.9)	60 (12.0)	
Day dysfunction due to sleep				
Never	416 (18.6)	329 (19.4)	82 (16.4)	0.090
<once a week	1,141 (51.2)	881 (51.8)	247 (49.5)	
1-2 times per week	544 (24.4)	398 (23.4)	133 (26.7)	
≥3 times per week	129 (5.8)	92 (5.4)	37 (7.4)	
Sleep efficiency (%)				
≥85	1,555 (69.7)	1,178 (69.3)	358 (71.8)	0.695
75–84	323 (14.5)	252 (14.8)	66 (13.2)	
65–74	158 (7.1)	117 (6.9)	35 (7.0)	
<65	194 (8.7)	153 (9.0)	40 (8.0)	
Sleep medicine during past month				
Never	2,040 (91.5)	1,550 (91.2)	462 (92.6)	0.356
<once a week	116 (5.2)	91 (5.4)	23 (4.6)	
1-2 times per week	57 (2.5)	43 (2.5)	13 (2.6)	
≥3 times per week	17 (0.8)	16 (0.9)	1 (0.2)	
Sleep quality				
No	1,055 (47.3)	820 (48.2)	220 (44.1)	0.103
Yes	1,175 (52.7)	880 (51.8)	279 (55.9)	

*P value from Chi-square test for categorical variables or from Student's *t*-test for continuous variables.

**Numbers may not add up due to missing data for selected variables.

**Table 3:** Odds ratio (OR) and 95% confidence intervals (CI) for poor sleep quality.

Characteristic	Unadjusted OR (95% CI)	Age and sex adjusted OR (95% CI)	Multivariate *adjusted OR (95% CI)
Sex			
Male	(1.00) Reference	(1.00) Reference	(1.00) Reference
Female	1.18 (0.97–1.44)	1.25 (1.02–1.55)	1.37 (1.08–1.73)
Smoking status			
Never	(1.00) Reference	(1.00) Reference	
Ever	1.27 (0.81–1.97)	1.32 (0.84–2.07)	
Number of alcohol drinks			
<1 drink/month	(1.00) Reference	(1.00) Reference	
1–19 drinks/month	1.05 (0.82–1.33)	1.06 (0.83–1.36)	
≥20 drinks/month	2.16 (0.94–4.96)	2.12 (0.92–4.91)	
Khat consumption			
No	(1.00) Reference	(1.00) Reference	
Yes	1.31 (0.98–1.75)	1.32 (0.98–1.77)	
Any caffeine containing beverages consumption			
No	(1.00) Reference	(1.00) Reference	(1.00) Reference
Yes	1.29 (1.05–1.59)	1.29 (1.04–1.59)	1.23 (0.99–1.53)
Physical activity			
No	(1.00) Reference	(1.00) Reference	(1.00) Reference
Yes	1.27 (1.05–1.54)	1.28 (1.05–1.55)	1.27 (1.02–1.64)

*Adjusted odds ratios provided for variables selected in the final model using forward logistic regression.

**Poor sleep quality: PSQI Global score > 5.

**Table 4:** Consumption of caffeinated beverages and khat use according to sleep quality.

Exposure	Poor sleep quality **N = 1,055	Good sleep quality **N = 1,175	*P* value
	*n* (%)	*n* (%)	
Any coffee containing beverages			
No	207 (17.7)	229 (21.75)	
Yes	965 (82.3)	824 (78.2)	0.015
Type of caffeinated beverage			
* Spris (coffee mixed with tea) *	*369 (31.4) *	*340 (32.2) *	*0.677 *
* Coffee/Espresso *	*588 (50.0) *	*402 (40.9) *	*<0.001 *
* Diluted coffee * ^ †^	*235 (20.0) *	*247 (23.4) *	*0.051 *
* Coke/Pepsi *	*463 (39.4) *	*426 (40.4) *	*0.639 *
Khat consumption			
No	904 (88.1)	847 (90.7)	
Yes	122 (11.9)	87 (9.3)	0.065
Khat consumption frequency/week			
0	923 (89.9)	865 (92.6)	
1-2	53 (5.2)	36 (3.9)	0.106
*≥3 *	*50 (4.9) *	*33 (3.5) *	

*P value from Chi-Square test for categorical variables or from Student's *t*-test for continuous variables.

^†^ *Diluted coffee includes macchiato and cappuccino*.

**Numbers may not add up due to missing data for selected variables.

**Table 5:** Prevalence and odds ratios for sleep quality parameters in relation to stimulant drinks and lifestyle characteristics.

		Short sleep duration		Long sleep latency		Day dysfunction		Poor sleep efficiency		Sleep medicine	
Sleep quality parameters		(≤6 hrs)		(>30 min)		due to Sleep		(<85%)		use	
	All (*n* = 2,230)	(*n* = 979)		(*n* = 1,082)		(*n* = 673)		(*n* = 675)		(*n* = 74)	
	*n*	%	OR (CI)	%	OR (CI)	%	OR (CI)	%	OR (CI)	%	OR (CI)
Smoking status											
Never	2,146	44.2	(1.00) Reference	48.0	(1.00) Reference	30.4	(1.00) Reference	29.8	(1.00) Reference	3.1	(1.00) Reference
Ever	84	38.1	0.67 (0.43–1.05)	61.9	1.68 (1.06–2.63)	25.0	0.79 (0.46–1.31)	42.9	1.74 (1.11–2.73)	8.3	2.84 (1.26–6.43)
Alcohol consumption											
<1 drink/month	1,900	44.8	(1.00) Reference	47.7	(1.00) Reference	30.1	(1.00) Reference	29.5	(1.00) Reference	3.0	(1.00) Reference
1–19 drinks/month	303	39.6	0.82 (0.64–1.05)	53.1	1.21 (0.95–1.55)	30.0	1.06 (0.81–1.39)	34.3	1.25 (0.96–1.62)	3.3	1.09 (0.55–2.16)
≥20 drinks/month	27	37.0	0.81 (0.37–1.81)	55.6	1.24 (0.57–2.71)	40.7	1.56 (0.70–3.47)	40.7	1.47 (0.66–3.24)	22.2	9.25 (3.53–24.20)
*P* value for trend			0.121		0.119		0.355		0.058		0.003
Khat consumption/week											
0	2,056	44.2	(1.00) Reference	46.9	(1.00) Reference	29.3	(1.00) Reference	29.0	(1.00) Reference	2.8	(1.00) Reference
1-2	89	37.1	0.77 (0.49–1.20)	56.2	1.29 (0.84–2.01)	25.8	0.93 (0.57–1.53)	34.8	1.29 (0.82–2.05)	5.6	2.03 (0.77–5.361)
≥3	85	43.4	1.05 (0.67–1.64)	61.5	1.72 (1.09–2.71)	32.7	1.28 (0.79–2.07)	44.6	1.91 (1.22–3.00)	10.8	4.42 (2.06–9.47)
*P* value for trend			0.777		0.011		0.420		0.003		<0.001
Any caffeine containing beverages consumption										
No	436	43.7	(1.00) Reference	41.1	(1.00) Reference	27.1	(1.00) Reference	27.7	(1.00) Reference	2.9	(1.00) Reference
Yes	1,789	44.0	1.00 (0.81–1.23)	50.3	1.48 (1.19–1.83)	30.9	1.21 (0.95–1.53)	30.8	1.15 (0.91–1.45)	3.4	1.15 (0.63–2.13)
Physical activity											
No	607	45.7	(1.00) Reference	43.3	(1.00) Reference	30.3	(1.00) Reference	26.5	(1.00) Reference	2.3	(1.00) Reference
Yes	1,495	43.7	0.95 (0.78–1.15)	50.4	1.29 (1.07–1.57)	30.1	1.00 (0.81–1.23)	31.9	1.27 (1.03–1.58)	3.8	1.60 (0.88–2.91)

*Adjusted for age and sex.

**Numbers may not add up due to missing data for selected variables.
